# Event Visualization and Trajectory Tracking of the Load Carried by Rotary Crane

**DOI:** 10.3390/s22020480

**Published:** 2022-01-09

**Authors:** Dawid Cekus, Filip Depta, Mariusz Kubanek, Łukasz Kuczyński, Paweł Kwiatoń

**Affiliations:** 1 Department of Mechanics and Machine Design Fundamentals, Faculty of Mechanical Engineering and Computer Science, Czestochowa University of Technology, Dąbrowskiego 73, 42-201 Częstochowa, Poland; dawid.cekus@pcz.pl; 2 Department of Computer Science, Faculty of Mechanical Engineering and Computer Science, Czestochowa University of Technology, Dabrowskiego 73, 42-201 Częstochowa, Poland; filip.depta@pcz.pl (F.D.); mariusz.kubanek@icis.pcz.pl (M.K.); lukasz.kuczynski@icis.pcz.pl (Ł.K.)

**Keywords:** dynamic vision sensor, trajectory detection, load motion, simulation, object tracking

## Abstract

Tracking the trajectory of the load carried by the rotary crane is an important problem that allows reducing the possibility of its damage by hitting an obstacle in its working area. On the basis of the trajectory, it is also possible to determine an appropriate control system that would allow for the safe transport of the load. This work concerns research on the load motion carried by a rotary crane. For this purpose, the laboratory crane model was designed in Solidworks software, and numerical simulations were made using the Motion module. The developed laboratory model is a scaled equivalent of the real Liebherr LTM 1020 object. The crane control included two movements: changing the inclination angle of the crane’s boom and rotation of the jib with the platform. On the basis of the developed model, a test stand was built, which allowed for the verification of numerical results. Event visualization and trajectory tracking were made using a dynamic vision sensor (DVS) and the Tracker program. Based on the obtained experimental results, the developed numerical model was verified. The proposed trajectory tracking method can be used to develop a control system to prevent collisions during the crane’s duty cycle.

## 1. Introduction

Devices for load transporting are one of the basic machines of almost every industrial plant or construction site. These machines, which include a rotary crane, allow for the safe handling of loads with a limited range of work. Along with the increase in the technological process, devices are designed to enable the transfer of larger loads with the simultaneous optimization of the structure. The problem of load motion analysis is currently one of the most important problems in research on transporting devices. Determining the behavior of the carried load during its transport provides for the development of more efficient machine control systems. In addition, it is also possible to prevent dangerous collisions that may lead to machine or load damage [1,2].

In the literature of the subject, one can mainly find works based on analytical models [3,4,5,6,7,8,9]. However, in the case of designing new devices, the use of CAD/CAE software to conduct numerical simulations is indispensable, especially during the strength analysis [10,11]. Due to certain limitations imposed by such programs, it is necessary to verify the developed numerical models by performing experimental tests by tracking the load. One of the methods of tracking the transported load is event-based detection by using a dynamic vision sensor (DVS). The advantage of this method is that, unlike conventional cameras, they measure intensity over a longer period of time and record changes of even single pixels [12,13]. Event-based sensors, apart from detecting the area of machine operation, are also used in human–machine interactions [14,15,16] or during fluid mechanics experiments [17,18,19].

In the case of tracking the working area of a transporting machine, conventional cameras are most often used. The paper [20] presents a method for determining the deflection angle of a load carried by an overhead crane. This method consisted in tracing the marker using the mean shift algorithm and the Kalman filter. Finally, the angle of deflection of the load is calculated using the geometric method and the angle plot. The work [21] presents visualizations of the crane’s working environment with the use of laser-scanned point clouds and vision. The approach enables real-time tracking of the work area and is dynamically updated. The proposed method was verified and validated during experimental tests on the object. The vision-based method was also used in [22] to monitor the deflections of the transferred load. In this case, special attention was paid to blind lifts. Due to limited visibility, blind lifts are one of the most dangerous crane operations. The conducted laboratory and field tests confirmed that the proposed technique is an appropriate tool supporting the operator’s work. The problem of the blind lift was also discussed in [23]. In this work, using a camera and encoders, basic parameters of the working area were determined, such as position and rotation of the load, angle of boom change, rope length, and location of workers. The developed system of multisensors made it possible to determine the load orientation, detection of passages, or warning against a collision. Moreover, the sensors used made it possible to recreate the 3D workspace in the form of a point cloud. Vision techniques allowing to increase the safety of cranes operation are also presented in the works [24,25,26]. The paper [24] presents the concept of developing a crane operation supervision system with the use of a camera system and specialized sensors. A method of determining the load deflection angle with the use of a laser line emitter was proposed. A new technique of active control of the correct positioning of the gantry crane is presented in [25]. Using the MVS (machine-vision system), a control system was proposed that allows for precise positioning. In order to minimize fluctuations in the transferred load, the energy storage function and coupling control law were used. The paper [26] presents the technique of obstacle detection and visualization of the transferred load. Using monocular vision and marker plate, the payload was tracked for different auto-centring control strategies. Automatic obstacle avoidance was designed and experimentally verified. Work [27] deals with the subject of real-time clustering and tracking with the use of event-based sensors. Kalman filters were applied to smooth the trajectories received by the sensors. The research was carried out on an industrial robot during the object manipulation task. Various shapes of clusters as well as different speeds were analyzed in the tests. The topic of tracking the trajectory of an industrial robot was also presented in [28]. Using vision sensors, a technique for grasping moving objects by the robot was developed. The position of a moving object is constantly updated via the particle filter. Based on the grasping hypotheses, the robot can follow or catch the object with minimal task space error. Numerical optimization is used to determine the task space error.

Due to the development of the event-based vision technique, DVS sensors are increasingly used in problems related to real-time motion analysis. The use of DVS in such analysis is presented in [29]. An innovative method of presenting the stream of events has been proposed, which enables the use of information about its dynamic component. Using the approximation of the 3D geometry and the parametric model, the algorithm determines the flow of events with motion compensation. Moreover, the authors also discussed cases of inconsistency in the time model in the case of very fast traffic. Collision prevention during quadrotor motion analysis with the use of pair of dynamic vision sensors was presented in [30]. With the use of probabilistic trackers, updated with each event, it is possible to track spherical objects in space. The trajectory was determined using the Kalman filter and the mixed state space. The collision can be predicted by error propagation techniques. Experimental tests confirm the correctness of the proposed technique. The paper [31] presents a novel algorithm of quick target tracking based on the consistency of events. The object in motion was determined by evaluating the consistency according to the distribution of the event. The results of experimental tests confirm that the proposed algorithm can accurately track small objects moving at high speed in real-time. DVS sensors were also used in [32] to track line structures in data streams. The proposed method consisted in determining the planes of events of DVS sensors and tracking them in time. Using the low latency of each sensor event, the position of the line was determined within a few microseconds for any point in time. The advantages of the silicon retina technique for space imaging in relation to traditional vision methods have been analyzed in the article [33]. The research focused on the analysis of space objects. The capabilities of DVS sensors were demonstrated during field trials of telescopes. In addition, two event-based sensor architectures were validated.

**Figure 1:**
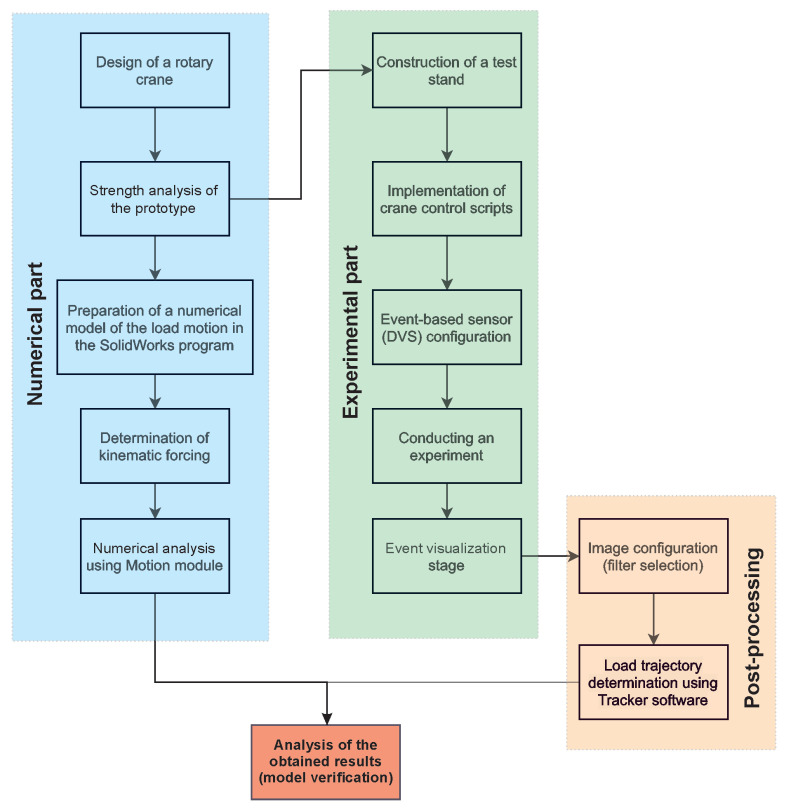
Flowchart of the completed tasks divided into numerical and experimental part, post-processing and results analysis.

This paper presents an analysis of the motion of the load carried by the designed rotary crane. The simulations were performed in SolidWorks software using the Motion package. The results of numerical simulations were verified through experimental tests on a specially built research facility. Tracking the load during the experiment was completed by visualizing the events through a DVS sensor. On the basis of the obtained visualization, the trajectories of the carried load were determined in the Tracker program. The obtained results confirm that the numerical model has been correctly developed. The individual stages of the activities performed in the article are presented in Figure 1. The innovation of this article is both the design of a laboratory rotary crane equipped with electric devices and the application of dynamic vision sensors to verify the obtained numerical calculations of load motion.

## 2. Laboratory Crane Model

The rotary crane, which was designed for this work, was based on the design of the Liebherr 1020 LTM truck crane. During the design process, the motion of the crane was limited to four characteristic movements: rotation of the platform with the jib, change of the jib angle, change of the jib length and operation of the winch [34]. Due to the matter related to crane control, it was decided to replace hydraulic drives with electric ones. One of the major advantages of electric drive is its quick and precise response, which will allow for more efficient motion at the stage of developing an appropriate control system [35]. These motors, unlike hydraulics, provide high positioning accuracy and programmable stop positions, which allows for greater possibilities of machine control.

**Figure 2:**
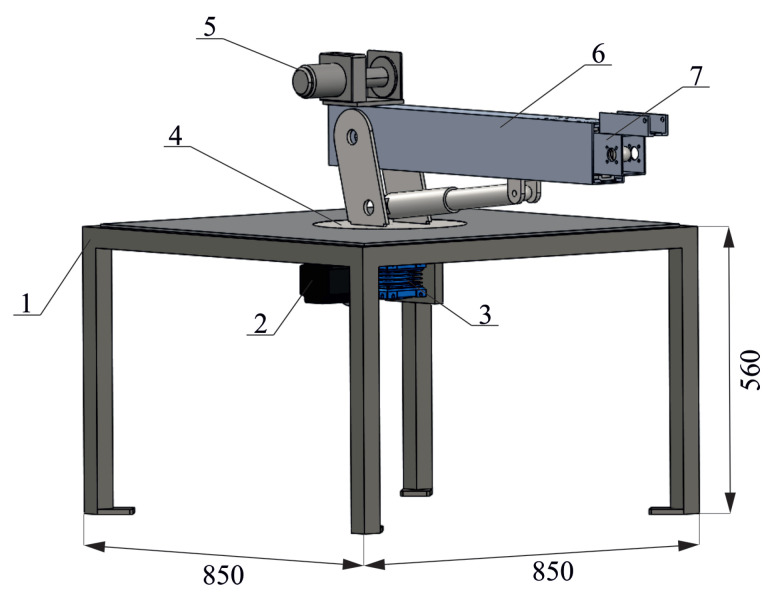
Schematic diagram of the designed laboratory crane (main components).

**Figure 3:**
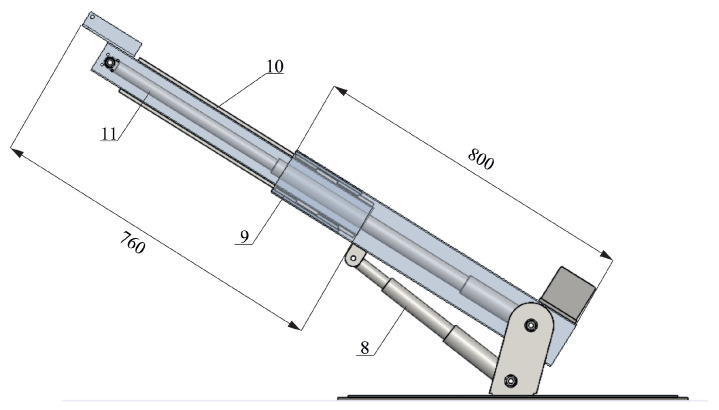
Schematic diagram of the designed laboratory crane (telescopic boom).

The rotary crane model was made in the SolidWorks software (Figure 2 and Figure 3). This model consists of:

- Crane chassis frame (1);
- Stepper motor (2);
- Worm gear (3);
- Thrust bearing (4);
- Winch (5);
- Aluminum profiles (6 and 7) constituting a telescopic boom;
- Actutator for changing boom inclination angle (8);
- Linear trolleys (9);
- Rails (10);
- Actuator for changing the length of the boom (11).

In the model, the additional support system was not considered—the duty cycle of the laboratory crane can only correspond to cases where the rotary crane rests on its wheels. The change in the length of the telescopic boom and the change in the angle of inclination was obtained with the use of linear actuators (8 and 11). The rotation of the platform with the jib is carried out using the stepper motor (2) connected with a worm gear (3). In order to obtain the expected smoothness and telescoping precision, linear guides were installed in the boom members (6 and 7), constituting a combination of linear trolleys (9) and rails (10). The dimensions of the aluminium profiles and the frame, as well as the maximum crane capacity, were selected using a strength analysis performed in the Simulation module of SolidWorks. The results of the static analysis have not been shown in the paper because they are not significant in terms of the scope of the manuscript.

The numerical model did not take into account the standardized elements, because their influence on the results of both the strength and motion analysis may be neglected.

The analysis of the motion of the load carried by the rotary crane was conducted using the Motion module of the SolidWorks package. The Motion Analysis study provides complete, quantitative information on the kinematics and dynamics of all components of a moving object [36]. The duty cycle simulation is performed using the CAD model and the constraints adopted during creating the assembly [1].

## 3. Numerical Research

**Figure 4:**
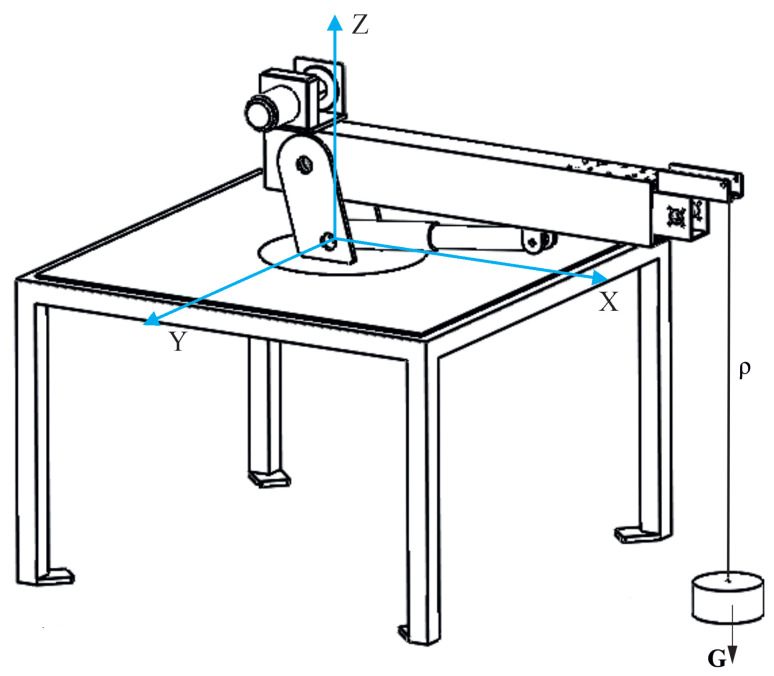
Numerical model.

In the numerical tests (Figure 4), it was assumed that the crane rests on the non-deformable ground and that it is not subject to any kinematic forces during $t_{0}$ (initial time). The interaction between the supports and the ground was achieved through linear springs.

**Figure 5:**
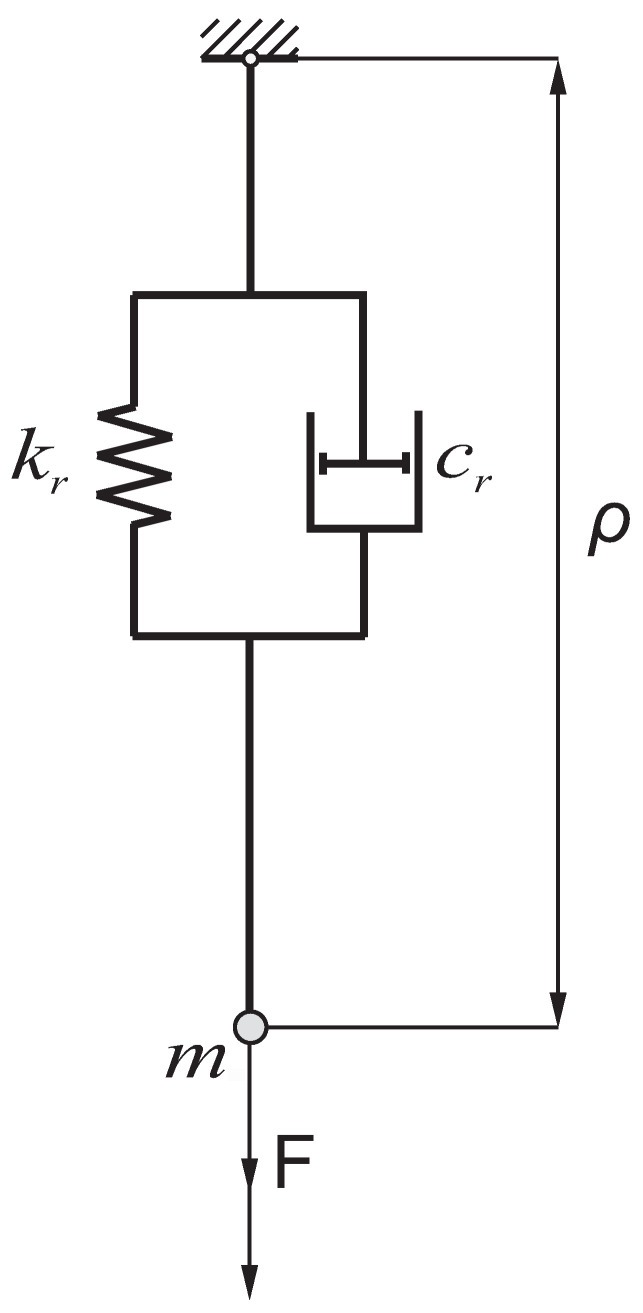
Graphical representation of rope modeling in the SolidWorks program.

Due to the limitations of the SolidWorks program, the rope (ρ) has been modeled as a combination of a linear spring and a damper (Figure 5). The length of the spring is determined by the program based on the values of the stiffness and/or damping coefficients. The rope stiffness and damping coefficients were, respectively, $k_{r}=1.102\cdot 10^{7}$ N/m, $c_{r}=2.09\cdot 10^{4}$ kg/s. Coefficients were determined according to the formulas [37]:

(1) $$k_{r}=\frac{E_{r}A_{r}}{L_{r}},$$

(2) $$c_{r}=2\nu_{r}\sqrt{km},$$

where: $E_{r}$—Young’s modulus of the rope material; $A_{r}$—rope cross-sectional area; $L_{r}$—current rope length; $\nu_{r}$—dimensionless damping factor; and *m*—mass of carried load.

The working cycle of the laboratory crane included two stages of work: change of the inclination angle and rotation of the platform along with the jib. For the purposes of numerical simulations, the length of the rope ρ = 0.7 m was assumed.

**Figure 6:**
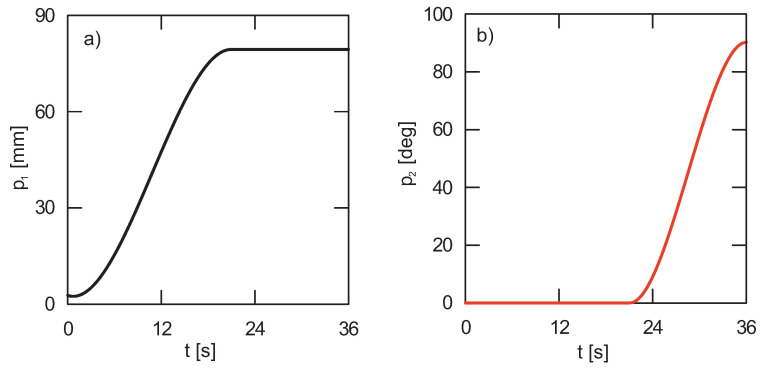
Kinematic forcing—(**a**) inclination angle actuator, (**b**) platform rotation.

Kinematic constraints (Figure 6) have been implemented into the model in the form of displacement segments of appropriate members. Based on the implemented displacements, the SolidWorks program calculates velocities and accelerations, respectively. Cubic interpolation was selected as a function of the default segment type [1].

For the purposes of the load movement analysis, the following assumptions were made:

- The crane is placed on a stable, non-deformable ground;
- The platform rotation angle with the jib is in the range from 0${}^{\circ }$ to 270${}^{\circ }$;
- The effect of wind pressure was omitted in the model;
- Due to the limitations of the program, the fixed length of the rope on which the load was suspended was assumed;
- The problem of friction force in joints was omitted.

**Figure 7:**
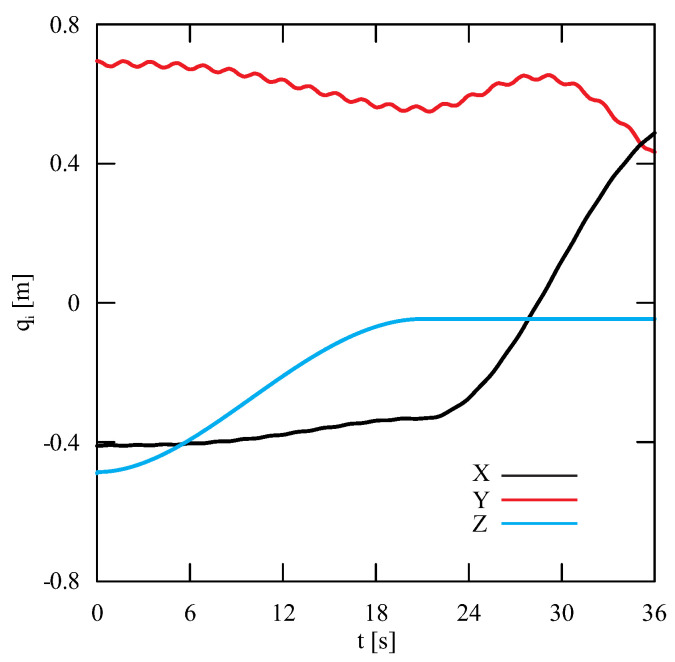
Change of generalized coordinates as a function of time.

**Figure 8:**
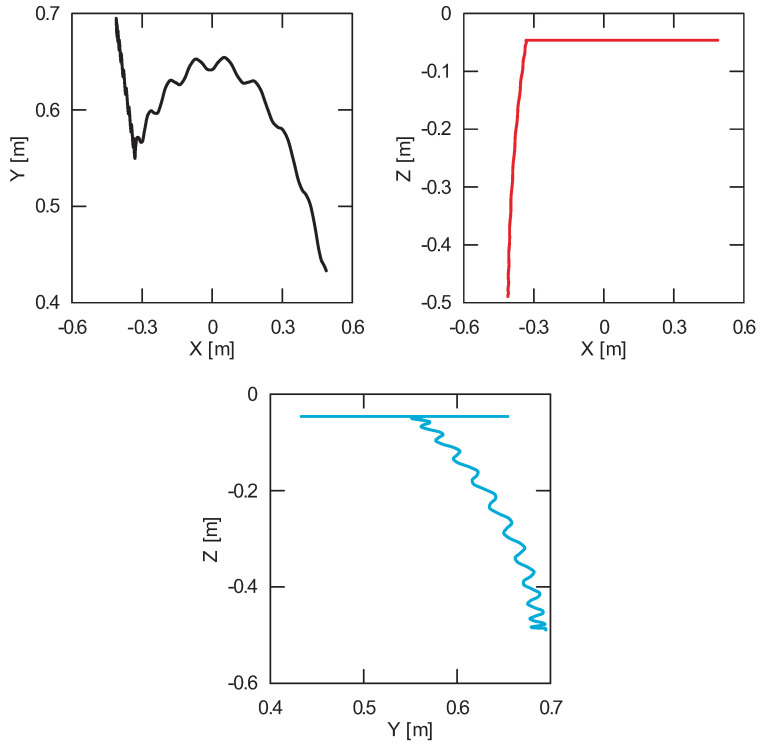
Projections of the trajectory of the carried load on the rotation and lifting planes.

Based on the numerical analysis, the course of the generalized coordinates as a function of time (Figure 7) and projections of the trajectory of the carried load in the lifting and rotation planes were obtained (Figure 8). Accurate determination of the coordinates of the carried load is useful especially during studies on the stability analysis of transporting machines. The use of numerical analysis in load tracking is an important aspect during the facility design phase because design errors can be detected while reducing costs. However, to determine whether the numerical model has been properly developed, it is necessary to verify it.

## 4. Experimental Detection and Tracking of the Load

### 4.1. Test Stand

**Figure 9:**
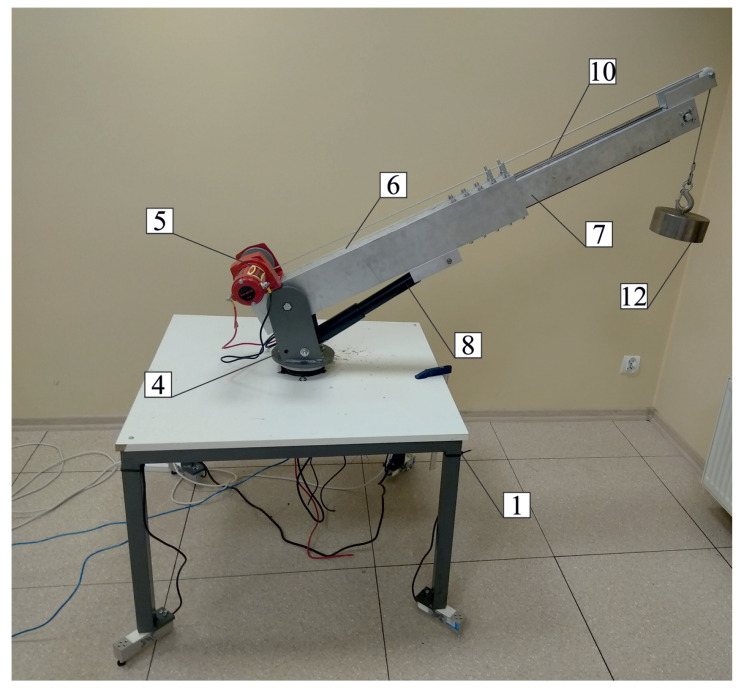
Laboratory truck crane: 1—chassis frame; 4—thrust bearing; 5—winch; 6; 7—aluminum profiles; 8—inclination angle actuator; 10—rail; 12—load.

On the basis of the developed model, a test stand for a laboratory crane was built (Figure 9), which allowed for the verification of the numerical results from the previous section. The stand, apart from the elements presented in Section 2, also consisted of two power supplies with a power of 750 W and 2 kW, motor drivers and two Arduino boards based on AVR ATmega328P microcontrollers. Arduino boards were responsible for movement of individual members.

The operation of the laboratory crane is controlled by the Arduino platform, which is responsible for sending signals to the actuators’ controllers. The signals are generated by specially developed scripts implemented in the memory of the microcontroller. The scripts contain the times of switching on and off of individual motors and their maximum speeds in the form of pulses per second, collected in tabular form. This method of control allows for the reproduction of the same working cycle as it was in the case of numerical analyzes. Additionally, scripts allowing the operator to control the crane in real-time have been prepared.

The initial positions of the laboratory crane members during the experimental tests corresponded to the positions in numerical simulation, that is, the inclination change actuator and the boom length change actuator remained folded, while the boom was angled 40${}^{\circ }$ to the X axis. To adjust the speeds of the drives during the experiment according to the control functions presented in the previous chapter, the speeds obtained from the entered kinematic inputs in the numerical model were implemented in the memory of the microcontroller.

### 4.2. Experimental Tests

The trajectory of the carried load was observed by the Dynamic Vision Sensor (DVS). The sensor is made in CMOS, 0.18 μm technology, where the fill factor is 25%. It is able to generate only contrast detection (CD) events [38]. Currently, the DVS sensor applications and use cases are for, e.g., feature detection and tracking, optical flow estimation, 3D reconstruction, pose estimation and SLAM, image reconstruction, motion segmentation, recognition, and neuromorphic control [39]. Detailed information on calibrating dynamic vision sensors, such as coarse time synchronization or estimating relative rotation, is described in the article [40].

**Figure 10:**
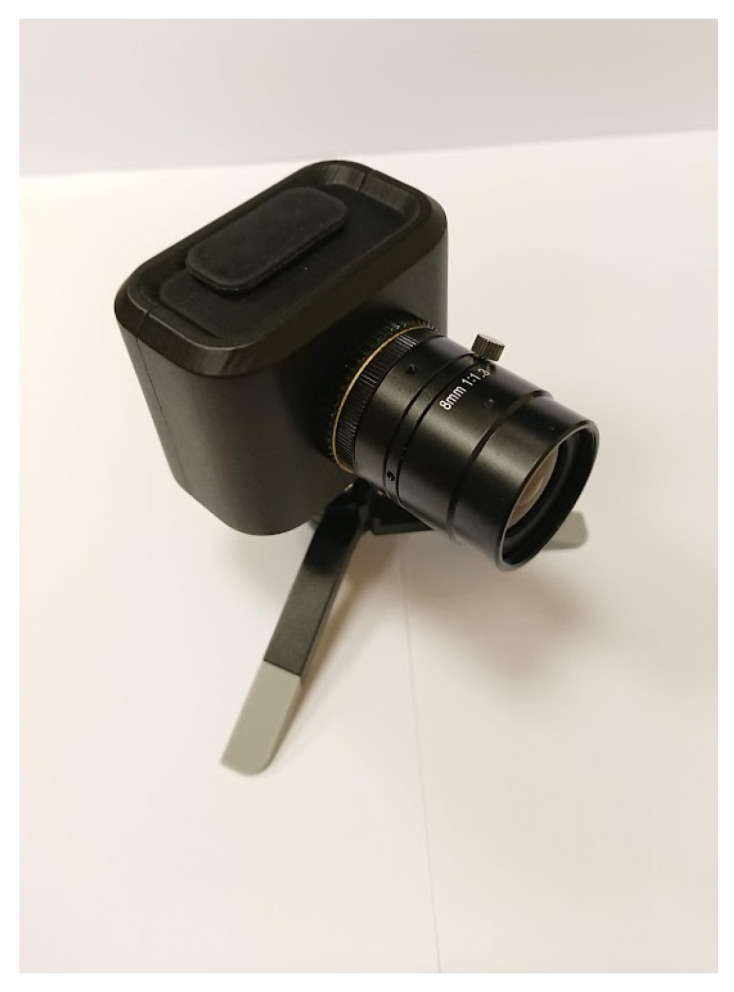
Dynamic vision sensor.

Classical vision sensors such as CMOS or CCD matrices, synchronously (according to a clock) generate whole frames of values. This method of vision acquisition was known, used, and studied for decades. Unfortunately, the frame-based vision has some disadvantages, e.g., high data redundancy, high bandwidth demand in short-latency use-cases, or limited dynamic range [41]. The DVS (see Figure 10), sometimes called “silicon retina” [41,42], functions differently. Each pixel of the sensor operates separately and elicits its events immediately when pixel illuminance changes [43]. Each event receives a precise timestamp (pixel location/address is also known). There are two types of events—“on” and “off”. “On” event occurs when light illuminance increases and “off” event contrary. Static scene objects (with static illumination) are almost invisible. Such kind of object separation could enhance trajectory tracker performance. According to Prophesee’s VGA evaluation kit briefing document, the sensor latency is between 40 and 200 μs. Moreover, even if the evaluation kit interface or host machine generate more latencies (due to interface or OS processes), the events are timestamped with microsecond precision, so the flow of the events can be precisely reconstructed [38]. The standard camera framerate is between 25 and 60 FPS. For the highest FPS, it is more than 16 ms of latency, for light changes occurred at the frame begging, so the latency for raw events, not converted to a video file—due to timer triggered events (frames)—is incomparable.

For the experiment, there was a Prophesee Evaluation Kit mounted on a tripod. The kit device is equipped with a 3rd generation VGA CIS Metavision^®^ sensor. It has a resolution of 640 × 480 px^2^, a dynamic range of more than 120 dB, and a C-mount socket for the lens. The communication and the power supply, are realized over the USB 3.0 interface, connected to a host device (e.g., PC), which can directly visualize, analyze, or record the events stream. The sensor was used with a kit lens, which has 70° D-FOV.

During a recording session, events streams from the DVS were recorded with Metavision Player software from Prophesee. In order to reduce background noise, the sensor biases (settings) were experimentally tuned before the recording session. Two parameters have been tuned (diff_on and diff_off) and correspondingly set to 404 mV and 208 mV. After each recording, the events stream visualization was rendered as a standard video file with a framerate of 25 fps. Due to slow target movement, events accumulation time per frame has been set to 200% (which corresponds to 2 frames time), to enhance target shape and visibility.

**Figure 11:**
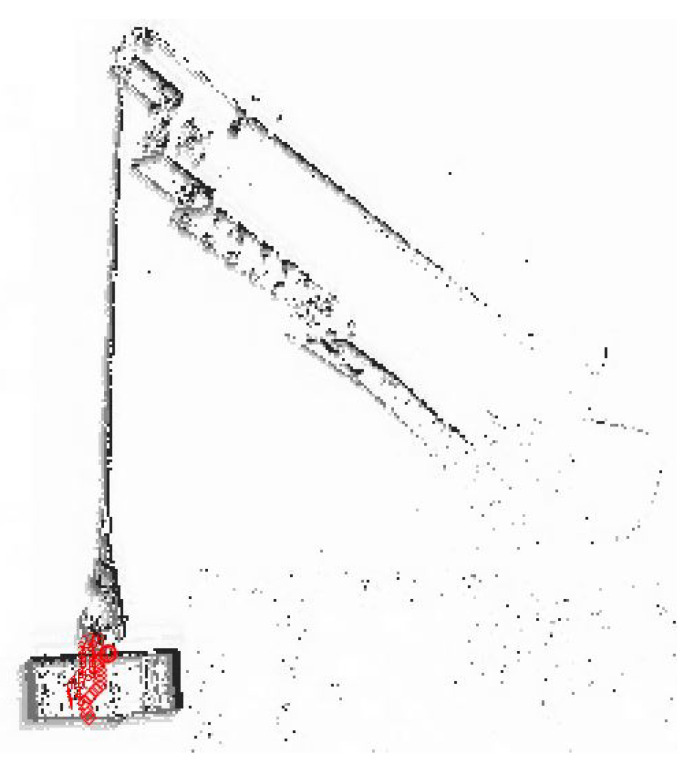
The process of tracking the point in the Tracker program from the received event visualization.

After the event visualization stage, the Tracker Video Analysis and Modeling Tool software was used to determine the trajectory of the transferred load (suspension point). This program is a tool enabling video analysis and modeling. It is built on the Java Open Source Physics (OSP) platform and is intended for use in physics education [44]. Based on the predefined template, the program is able to track the pattern change and save the coordinates relative to the specified coordinate system (see Figure 11). In order to increase the accuracy, it is also necessary to define calibration tools and use filters built into the program. These filters allow one to correct the colour of the video, which results in a more precise sample determination.

## 5. Discussion

**Figure 12:**
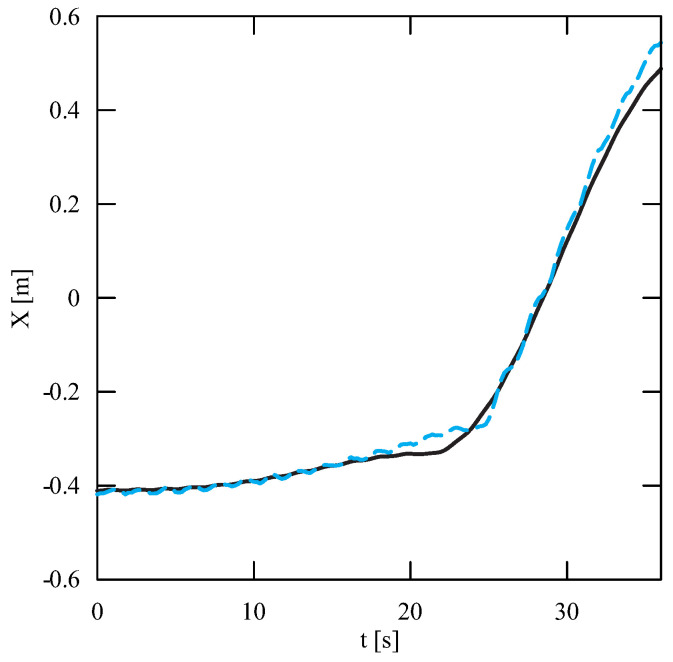
Change of the X coordinate as a function of time (solid line—numerical calculations, dashed line—experiment results).

**Figure 13:**
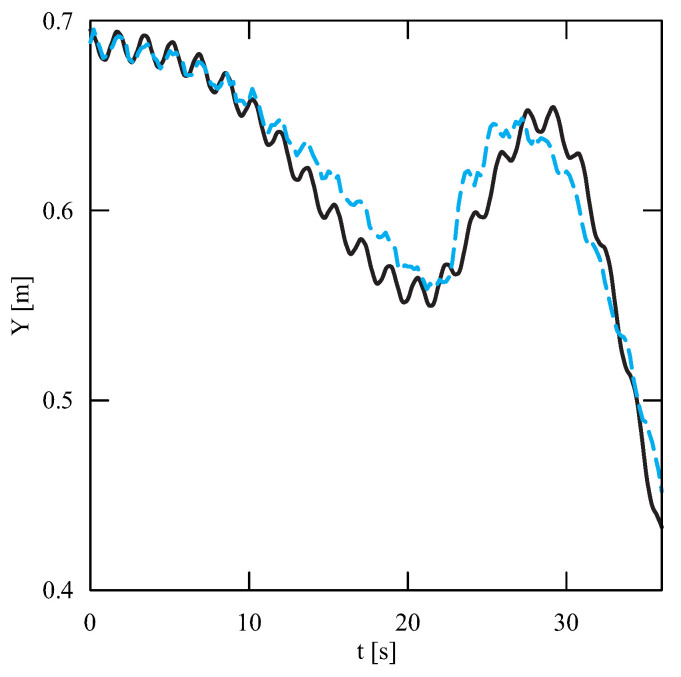
Change of the Y coordinate as a function of time (solid line—numerical calculations, dashed line—experiment results).

**Figure 14:**
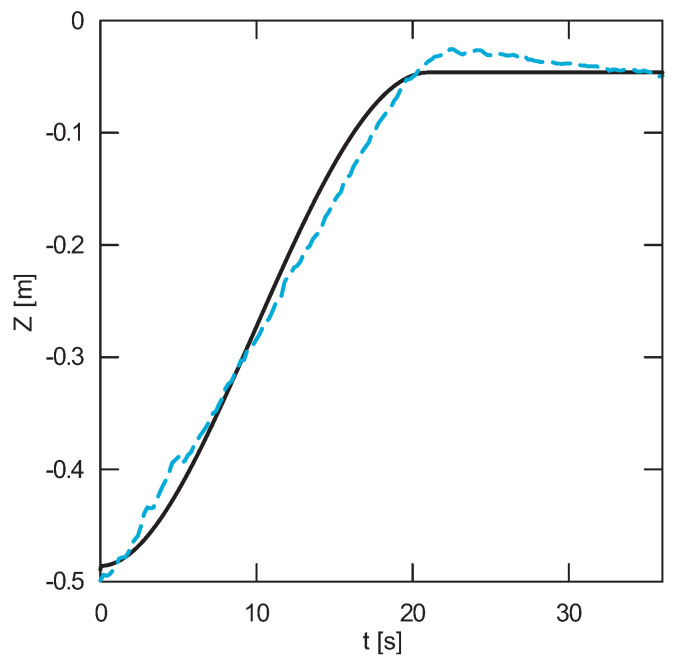
Change of the Z coordinate as a function of time (solid line—numerical calculations, dashed line—experiment results).

**Figure 15:**
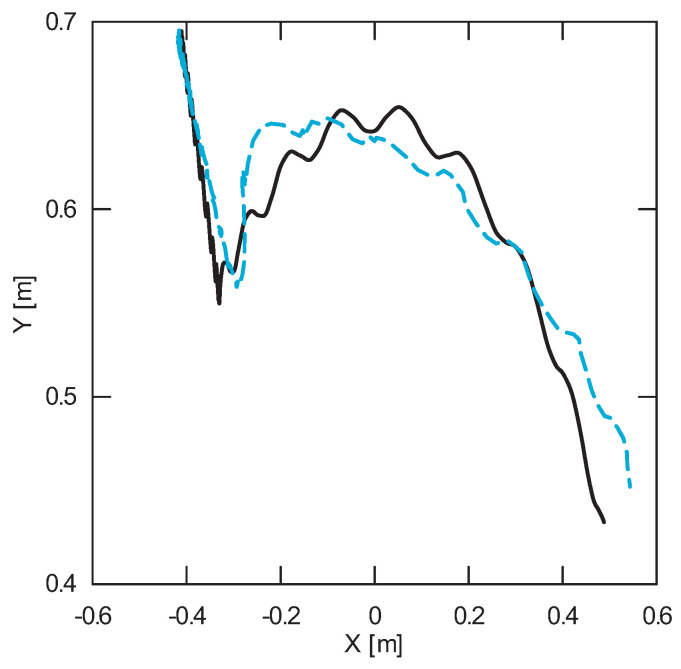
Projection of the trajectory of the transferred load in the rotation plane XY (solid line—numerical calculations; dashed line—experiment results).

**Figure 16:**
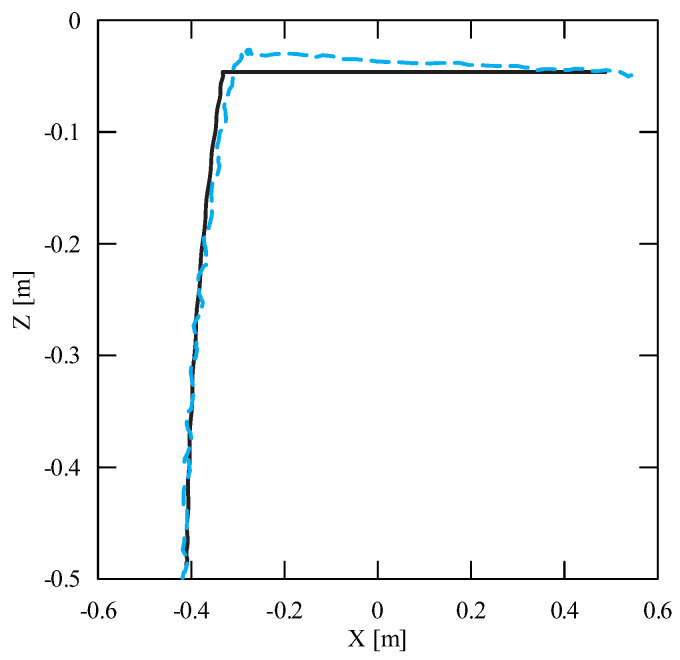
Projection of the trajectory of the transferred load in the lifting plane XZ (solid line—numerical calculations; dashed line—experiment results).

**Figure 17:**
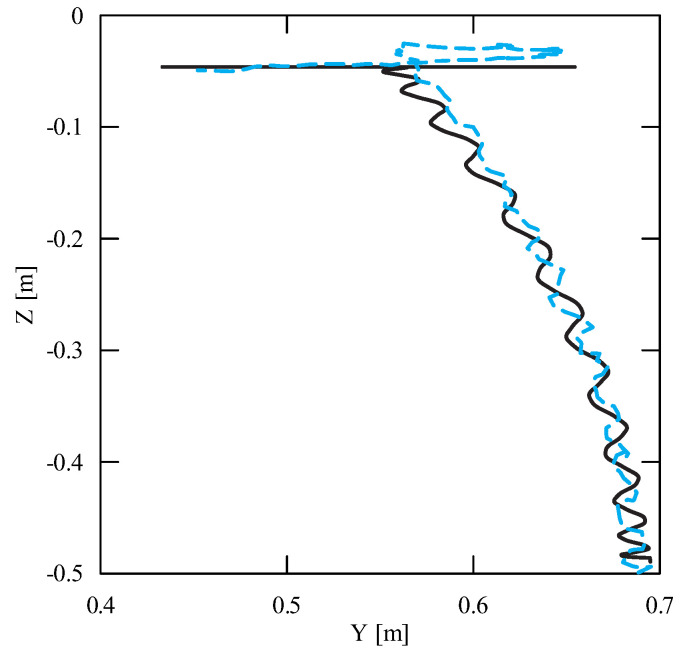
Projection of the trajectory of the transferred load in the lifting plane YZ (solid line—numerical calculations; dashed line—experiment results).

The obtained results of experimental tests show a similar trend of the coordinates changes of the transferred load as a function of time (Figure 12, Figure 13, Figure 14, Figure 15, Figure 16 and Figure 17). In experimental tests, smaller change of the coordinate during the inclination angle change stage is due to limitations imposed by the SolidWorks program. Due to limitations, the deformability of some elements (rope or load sling) and the influence of aerodynamic drag were neglected during the numerical analysis. Therefore, in the simulation results fluctuations are larger than in the experiment. By analyzing the projections of the trajectory of the carried load (Figure 15, Figure 16 and Figure 17), it can be concluded that the differences are more significant than in the case of the coordinates. Larger differences were observed both in the plane of rotation and in the plane of lifting.

**Figure 18:**
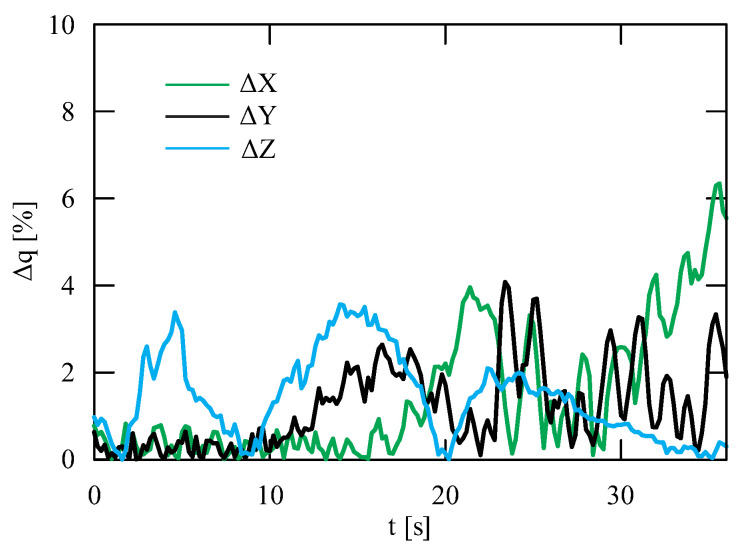
Percentage error of the generalized coordinates obtained during simulation and experimental tests.

By analyzing the percentage error (Figure 18) of the obtained numerical and experimental results, it can be clearly stated that the numerical model has been correctly developed. The biggest differences can be noticed during the final stage of the working cycle for the X coordinate. The maximum percentage error obtained during the tests was, respectively, for the coordinates X = 6.34%, Y = 4.07% and Z = 3.57%. The average percentage error for each coordinate did not exceed 1.5%, while the maximum coordinate error was 0.06 m. The biggest deviation of the Z coordinate was observed in the initial and final stages of the change of the inclination angle. In the case of the X and Y coordinates, the most visible discrepancy between the results can be found during the second stage of the working cycle—platform rotation. The reason for this disproportion is the principle of operation of a real object. During the rotation, as a result of the inertia force of the crane, it moves even slightly after the kinematic forcing ceases (as opposed to the numerical model). Therefore, it can be concluded that dynamic vision sensors can be successfully used during the stage of verification of numerical models analyzing the movement of the transported load.

## 6. Conclusions

This paper presents the use of event-based sensors in the verification process of the numerical model of load movement analysis. The rotary crane was designed based on the mechanical part of the truck crane. Hydraulic drives were replaced with electric drives. Numerical simulations of the analysis of the movement of the transported load were carried out in SolidWorks using the Motion module. The developed numerical model was verified by conducting experimental tests on the constructed real object. During the experimental tests, dynamic vision sensors were used to determine the trajectory of the carried load. Based on the event visualization and the Tracker program, the coordinate changes and trajectory projections were determined. By analyzing the obtained results, it can be concluded that the developed numerical model in the commercial SolidWorks program has been correctly formulated. The course of the generalized coordinates as a function of time during the experimental studies was similar to those obtained numerically. The biggest differences were noticed for the X coordinate (the error was 0.06 m) at the end of the duty cycle. This is due to a delay in the operation of the motors of the real object and the limitation of numerical software—it is not possible to take into account the deformability of individual elements and aerodynamic drag. In addition, greater differences were observed in perceived trajectory projections—both in the lift and rotation planes. Based on the obtained results, it can be concluded that dynamic vision sensors can be used in the process of tracking the real movement of the load. Their unquestionable advantage is tracking even fast-moving objects, which can be useful when detecting moving obstacles.

The next stage of work will be the development of a scripts that allows one to track the trajectory of the load without the need to visualize events and use additional software—the so-called event-based tracking. In addition, it is planned to develop a control system that will allow you to avoid obstacles in the movement located in the working area of the machine. In further works, it is also planned to conduct research on the influence of rotary crane speed on the deviations between the numerical and experimental results.

## Data Availability

Not applicable.
